# Polychlorinated Biphenyls Disturb Differentiation of Normal Human Neural Progenitor Cells: Clue for Involvement of Thyroid Hormone Receptors

**DOI:** 10.1289/ehp.7793

**Published:** 2005-04-18

**Authors:** Ellen Fritsche, Jason E. Cline, Ngoc-Ha Nguyen, Thomas S. Scanlan, Josef Abel

**Affiliations:** ^1^Group of Toxicology, Institut für umweltmedizinische Forschung gGmbH an der Heinrich-Heine Universität, Düsseldorf, Germany; ^2^Departments of Pharmaceutical Chemistry and Cellular and Molecular Pharmacology, University of California-San Francisco, San Francisco, California, USA

**Keywords:** NH-3, NHNP cells, oligodendrocyte, PCB, retinoic acid, thyroid hormone receptors

## Abstract

Polychlorinated biphenyls (PCBs) are ubiquitous environmental chemicals that accumulate in adipose tissues over the food chain. Epidemiologic studies have indicated that PCBs influence brain development. Children who are exposed to PCBs during development suffer from neuropsychologic deficits such as a lower full-scale IQ (intelligence quotient), reduced visual recognition memory, and attention and motor deficits. The mechanisms leading to these effects are not fully understood. It has been speculated that PCBs may affect brain development by interfering with thyroid hormone (TH) signaling. Because most of the data are from animal studies, we established a model using primary normal human neural progenitor (NHNP) cells to determine if PCBs interfere with TH-dependent neural differentiation. NHNP cells differentiate into neurons, astrocytes, and oligodendrocytes in culture, and they express a variety of drug metabolism enzymes and nuclear receptors. Like triiodothyronine (T_3_), treatment with the mono-*ortho*-substituted PCB-118 (2,3′,4,4′,5-pentachlorobiphenyl; 0.01–1 μM) leads to a dose-dependent increase of oligodendrocyte formation. This effect was congener specific, because the coplanar PCB-126 (3,3′,4,4′,5-pentachlorobiphenyl) had no effect. Similar to the T_3_ response, the PCB-mediated effect on oligodendrocyte formation was blocked by retinoic acid and the thyroid hormone receptor antagonist NH-3. These results suggest that PCB-118 mimics T_3_ action via the TH pathway.

Polychlorinated biphenyls (PCBs) are anthropogenic industrial chemicals, the production of which was banned in the 1970s because of their presumed carcinogenicity (Chana et al. 2002). However, these chemicals are still present in the food chain; they accumulate in animal and human tissues and are among the most abundant persistent organic pollutants found in humans (DeKoning and Karmaus 2000; Kim et al. 2004). Depending on their degree of chlorination, they are metabolized to their hydroxy- and/or sulfur-containing metabolites (Haraguchi et al. 1997). PCBs can cross the placenta, and infants are exposed via contaminated breast milk (DeKoning and Karmaus 2000).

Epidemiologic studies have indicated that PCBs influence brain development (reviewed by Schantz et al. 2003). Children who are exposed during development exhibit neuropsychologic deficits such as lower full-scale IQ (intelligence quotient), reduced visual recognition memory, and attention and motor deficits (Ayotte et al. 2003; Darvill et al. 2000; Huisman et al. 1995a, 1995b; Osius et al. 1999; Walkowiak et al. 2001). Results from studies in rodents supported these findings (Berger et al. 2001; Lilienthal et al. 1990; Roegge et al. 2000; Widholm et al. 2001). PCBs decrease circulating levels of thyroxine (T_4_) in animals (Brouwer et al. 1998; Gauger et al. 2004; Meerts et al. 2002). The neuropsychologic findings in offspring after developmental exposure to PCBs overlap with those described for maternal thyroid insufficiency (Haddow et al. 1999; Morreale et al. 2000; Pop et al. 1999). However, exposure at doses that lower serum thyroid hormone (TH) did not always produce signs of hypothyroidism [e.g., no elevation in TSH (Barter and Klaassen 1992; Kolaja and Klaassen 1998), no lowering of body weight of rat pups (Zoeller et al. 2000), and acceleration of eye opening in rat pups that can also be caused by high levels of TH (Goldey et al. 1995)].

Epidemiologic studies do not uniformly find an association between PCBs and thyroid homeostasis. A negative correlation between circulating levels of TH and PCB exposure and a positive correlation between the TH-regulating hormone thyrotropin (TSH) and PCB exposure have been observed (Osius et al. 1999; Schell et al. 2002). Others found no association between PCB exposure and disturbances of the TH pathway. This may be due to comparing combined high-and low-exposure groups to the reference group. Nevertheless, all observed hormone levels in these epidemiologic studies were within the normal range (Hagmar 2003) [i.e., accidental exposure to PCBs was not associated with overt hypothyroidism (Nagayama et al. 2001)].

Because there is no clear relationship between PCB exposure, blood TH levels, and symptoms of hypothyroidism in animals or in humans, several investigators have speculated that PCBs may affect brain development by directly interfering with TH signaling (McKinney and Waller 1998; Porterfield 2000; Porterfield and Hendry 1998). Dowling and Zoeller (2000) showed that RC3/neurogranin expression in the fetal rat brain is controlled by TH of maternal origin. This laboratory also demonstrated that the technical PCB mixture Aroclor 1254 regulated the TH-dependent genes myelin basic protein and RC3/neurogranin in a TH-like manner in animals (Zoeller et al. 2000). Thus, despite the anti-thyroid effect of PCBs on serum TH, they seem to act like TH at the cellular level.

On the basis of these findings and because no one has critically tested the hypothesis that PCBs can influence developmental events in the human brain, we asked two questions: *a*) Do PCBs have a TH agonistic/antagonistic effect on human neural development? and *b*) Which mechanisms are involved? For these purposes we established the model of normal human neural progenitor (NHNP) cells (Brannen and Sugaya 2000), which allow us to study the effect of these environmental chemicals on neural differentiation. Most studies on the effects of PCBs use Aroclor, which consists of many different PCB congeners. Rather than deal with a heterogeneous group, we chose two different specific PCB congeners: PCB-118, a compound with weak dioxin-like activity, and PCB-126, a congener with strong dioxin-like properties (van den Berg et al. 1998). We applied the single congener approach to identify specific PCB involvement in the disturbance of neural differentiation.

## Materials and Methods

### Chemicals.

Triiodothyronine (T_3_; Sigma-Aldrich, München, Germany) was diluted in ethanol at a concentration of 300 mM. *Ortho*-substituted PCB-118 (2,3′,4,4′,5-pentachlorobiphenyl), coplanar PCB-126 (3,3′,4,4′,5-pentachlorobiphenyl (both from Ökometric GmbH, Bayreuth, Germany), all-*trans*-retinoic acid (RA; Sigma-Aldrich) and the TH antagonist NH-3 (Nguyen et al. 2002) were diluted in DMSO (Sigma-Aldrich) at stock concentrations of 1.53, 1.59, 10, and 10 mM, respectively. Benzo(*a*)pyrene (BAP; Sigma-Aldrich) was diluted in tetrahydrofuran (10 mM).

### Cell culture and treatment.

NHNP cells were purchased from Cambrex BioScience (Verviers, Belgium) and cultured as neurospheres in NPMM (Neural Progenitor Maintenance Medium; Cambrex BioScience) at 37°C with 5% CO_2_. Medium was changed every 2–3 days. Upon significant growth (0.7-mm diameter), spheres were chopped with a McIlwaine tissue chopper as previously described (Svendsen et al. 1998); the resultant cubes formed new spheres within hours and were named according to increasing passage after each chopping event (passages 1–7).

For treatment of neurospheres, chemicals were diluted in NPMM to the following final concentrations: 30 nM T_3_; 0.01 μM, 0.1 μM and 1 μM PCB-118 and PCB-126; 10 μM BAP; 1 μM each RA and NH-3; and 0.065% DMSO. We treated 3–10 spheres with a diameter of approximately 0.4 mm each for 7 days before plating for differentiation. Spheres were treated with each chemical alone or with a cotreatment containing PCB-118 and either NH-3 or RA for 1 week. Differentiation of NHNP cells was initiated by growth factor withdrawal and plating onto poly-d-lysine coated chamber slides (BD Biosciences, Erembodegem, Belgium). Neurospheres were plated in a defined medium consisting of Dulbecco modified Eagle medium (DMEM)/F12 (3:1) supplemented with N2 (Invitrogen GmbH, Karlsruhe, Germany). After differentiating for 2 days, cells were fixed in 4% paraformaldehyde for 30 min and stored in phosphate-buffered saline (PBS) at 4°C until immunostaining was performed.

### Immunocytochemistry.

Fixed slides were washed two times for 5 min each in PBS. Slides were incubated with the following primary antibodies: *a*) double staining beta(III)tubulin 1:100 and glial fibrillary acidic protein (GFAP) 1:1,000 (both from Sigma-Aldrich) in PBS containing 0.3% Triton X-100, or *b*) mouse antioligodendrocyte marker O4 1:15 (Chemicon, Temecula, CA, USA) in PBS with 10% goat serum for 1 hr at 37°C followed by three 10-min washes with PBS. We used fluorescein isothiocyanate (FITC)- and/or Rhodamine Red-coupled secondary antibodies (1:100 each; Jackson ImmunoResearch, Dianova GmbH, Hamburg, Germany) for detection by incubating slides for 30 min at 37°C, followed by three 10-min washes with PBS. In the third wash, we added 0.1 μg/mL Hoechst for nuclear staining. After brief drying, slides were mounted with Vectashield Mounting Medium (Vector Laboratories, Burlingame, CA, USA), covered with cover glass, and sealed with nail polish.

Slides were examined using a fluorescent microscope (Olympus, Hamburg, Germany), and photographs were taken with a ColorView XS digital camera (Olympus). We determined the number of O4-positive oligodendrocytes for each individual sphere by manual counting.

### Statistical analysis.

The counts were approximately lognormally distributed. Therefore, we used the geometric mean and the standard deviation of the geometric mean. The *t*-test was performed after logarithmic transformation of the values, and each treatment was compared to its respective control. The inhibition values were not logarithmically transformed.

### RNA preparation and reverse transcription polymerase chain reaction.

Total RNA was prepared from 10–15 pooled untreated and undifferentiated spheres (passages 0–2) using the Absolutely RNA Microprep Kit (Stratagene, La Jolla, CA, USA). Reverse transcription polymerase chain reaction (RT-PCR) was performed as previously described (Döhr et al. 1995). Sequences and annealing temperatures of the PCR primers are listed in Table 1. Fragments were separated on a 3% agarose gel containing ethidium bromide and visualized under ultraviolet light. We used a 100-bp marker (peqlab, Erlangen, Germany) to estimate the appropriate sizes of the PCR fragments.

## Results

### Cultivation and molecular characterization of NHNP cells.

**Figure 1:**
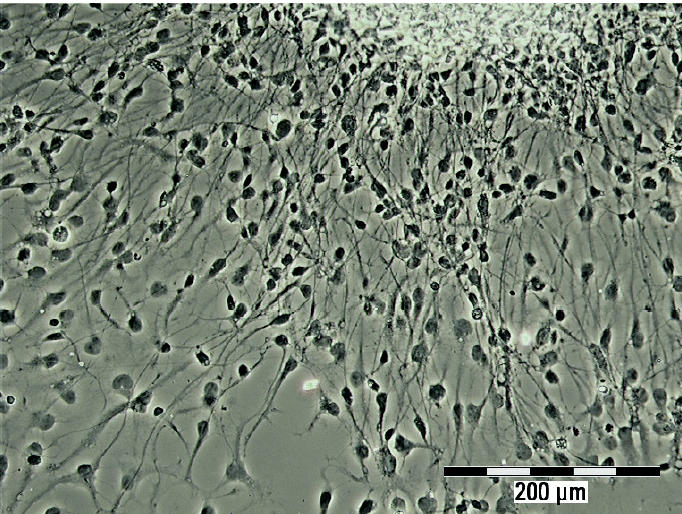
Neurosphere plated on poly-d-lysine–coated slides showing differentiation and radial outgrowth of cells out of the sphere after 4 days in culture. Phase contrast image. Bar = 200 μm.

**Figure 2:**
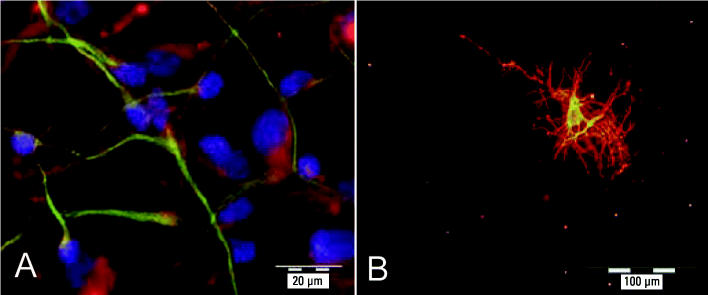
Immunocytochemical staining of differentiated NHNP cells. (*A*) β(III)Tubulin-positive neurons (green) and GFAP-positive astrocytes (red); nuclei stained with Hoechst. (*B*) O4-positive oligodendrocyte.

Neurospheres were successfully kept in suspension culture over several months. When they exceeded 0.7 mm in diameter, they were passaged by chopping into 0.3-mm cubes. This passaging was performed up to seven times during the lifespan of the NHNP cells. Plating of spheres onto poly-d-lysine–coated chamber slides under withdrawal of growth factors resulted in quick radial outgrowth and differentiation of the cells (Figure 1). After immunostaining, the differentiated cells were identified as neurons, astrocytes, and oligodendrocytes (Figure 2). Furthermore, neurons seem to form a neuronal network.

**Figure 3:**
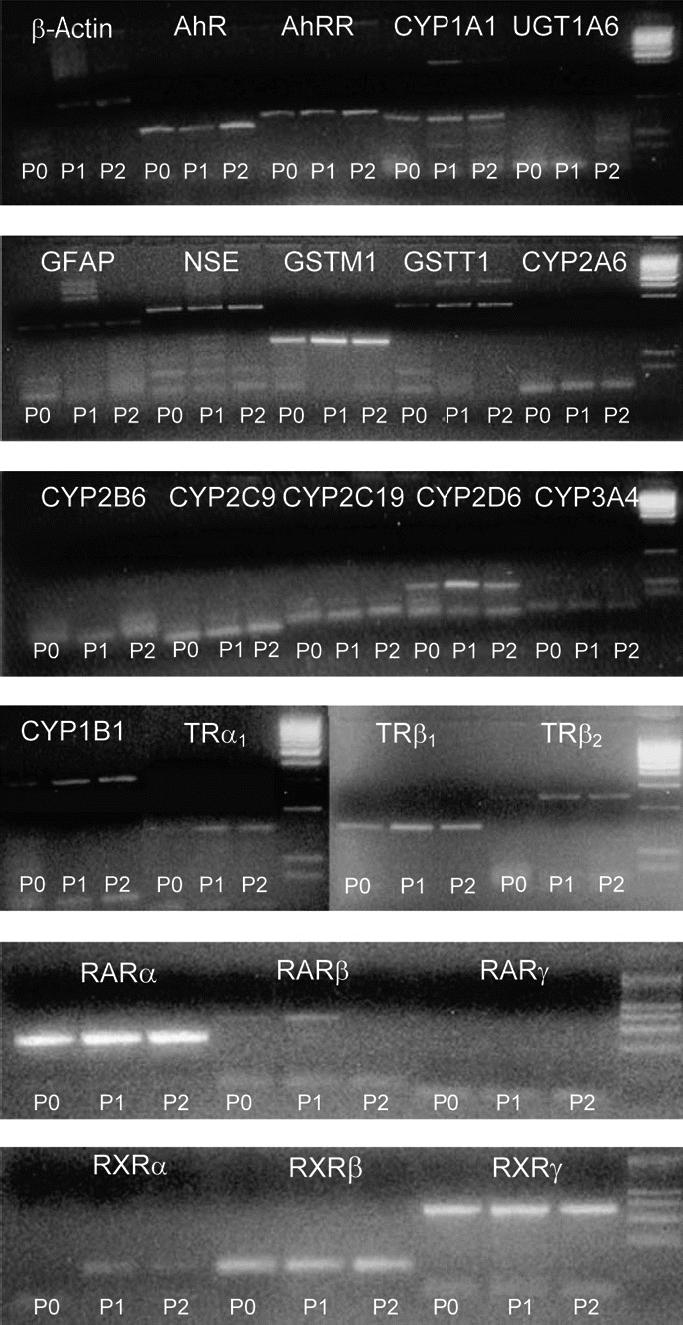
Expression patterns (RT-PCR) of different drug-metabolizing enzymes (CYPs, GSTs, UGT), neural markers (NSE, GFAP), and nuclear receptors (TRs, RARs, RXRs) during passaging of undifferentiated NHNP cells (P0–P2). RT-PCR was performed as previously described (Döhr et al. 1995). Respective primer sequences are given in Table 1. [The unspecific bands in some samples may be caused by the high cycle numbers (40) needed for detection of specific gene products due to the small amount of RNA obtained from each sample.]

To determine molecular characterization of NHNP cells we performed RT-PCRs of cell type–specific genes throughout the first three passages. We could identify typical gene products for the three different cell lineages in undifferentiated neurospheres: neuron specific enolase (*NSE*) for neurons, *GFAP* for astrocytes (Figure 3), and proteolipid protein with its splicing variant dm20 (data not shown) for oligodendrocytes. Finding these cell-specific markers in undifferentiated cells implies that specific cell fate is determined before plating and differentiation of cells.

To ascertain if NHNP cells are suitable for neurotoxicologic studies, we characterized them for their expression of genes playing a role in xenobiotic metabolism. The results obtained from undifferentiated neurospheres are shown in Figure 3. NHNP cells express the aryl hydrocarbon receptor (AhR) and the AhR repressor (AhRR), which represent central proteins in the regulation of AhR battery genes. Concerning phase 1 enzymes, we could detect gene products for cytochrome P450 (CYP)1A1, CYP1B1, and CYP2D6, whereas CYP2A6, CYP2B6, CYP2C9, CYP2C19, and CYP3A4 were not expressed. With regard to phase 2 enzymes, NHNP cells do express glutathione *S*-transferase (GST)M1 and GSTT1, but are abundant for UDP-glucuronosyltransferase (UGT)1A6. Hence, NHNP cells have the ability to metabolize xenobiotics.

Our objective was to investigate endocrine disruption of TH homeostasis in NHNP cells; thus we studied the expression of genes coding for thyroid hormone receptors (TR), retinoid acid (RAR), and retinoid X receptors (RXR), which are crucial molecules in hormone signal transduction. Undifferentiated NHNP cells express TRα_1_, β_1_, and β_2_, as well as RARα and β and RXRα, β, and γ. Therefore they represent a suitable cell model for investigating thyroid hormone disruption.

### Effects of T_3_ and PCBs on NHNP cells.

**Figure 4:**
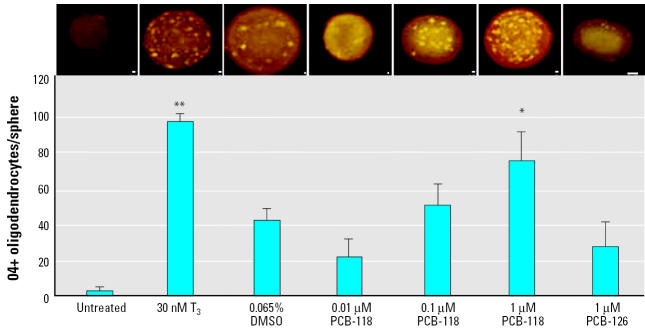
Induction of O4-positive (+) oligodendrocytes per sphere (geometric mean and SD) by T_3_ or PCB-118. Photographs show typical results of treatments (bars = 100 μm). Neurospheres were treated with T_3_ or PCBs as described in “Materials and Methods.” Values represent typical representatives of three independent experiments.
*p < 0.05, and **p < 0.01 by *t*-test.

Our initial goal was to investigate the mechanisms leading to disturbance of human brain development in a human *in vitro* model. Because disruption of thyroid hormone signaling is suspected to be involved in impairment of intellectual development by PCBs (reviewed by Zoeller and Crofton 2000) and because the timing of oligodendrocyte development seems to be dependent on TH (reviewed by Konig and Moura 2002), we investigated the occurrence of oligodendrocytes during differentiation of NHNP cells. Therefore, undifferentiated neurospheres were treated with 30 nM T_3_ for 1 week. After 2 additional days of differentiation, we found a significant increase in the number of oligodendrocytes formed compared to the medium controls (Figure 4). Treating neurospheres with PCB-118 for 1 week also led to an increase in oligodendrocyte formation, whereas PCB-126 had no effect. It is noteworthy that the solvent DMSO shows some intrinsic effect in this system (Figure 4). Thus, PCB-118 seems to have a TH-like effect in NHNP cells.

### Antagonism of T_3_ effects with RA and NH-3.

**Figure 5:**
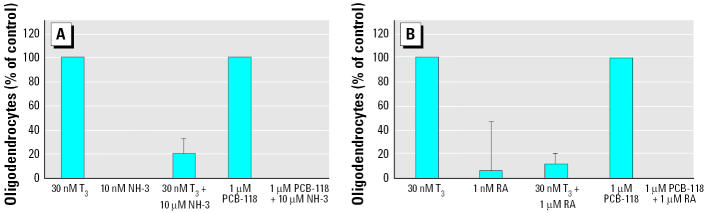
Antagonism of T_3_- or PCB-118-induced oligodendrocyte formation by (*A*) NH-3 and (*B*) RA. See “Materials and Methods” for details. Inhibitions are shown as a percentage of T_3_ or PCB-118 controls, respectively. Values represent typical representatives of three independent experiments.

To determine whether the TH-like effect of PCB-118 is mediated by TH receptors, we cotreated NHNP cells with 30 nM T_3_, 1 μM PCB-118, 1 μM RA, and 1 μM NH-3, or in combination. After 1 week, we counted the number of oligodendrocytes in the neurospheres. Both RA and NH-3 treatment prohibited the formation of oligodendrocytes by T_3_ and PCB-118 while having no intrinsic activity themselves (Figure 5). These results support the conclusion that PCB-118 acts by interfering with the TR complex.

## Discussion

It is now generally accepted that developmental exposure to drugs or chemicals can have adverse effects on the structure or function of the nervous system. Identification of such substances resulted mainly from epidemiologic data and animal studies. It is important to develop *in vitro* approaches because, in some cases, severe species differences can exist (Harry et al. 1998; Tilson 1996). In this article, we characterize an *in vitro* human neural model. To demonstrate the toxicologic usefulness of this model, we have shown the effects of two different PCB congeners on neural development. Although the ability of PCB congeners to induce cytochrome P450 enzymes has been intensively studied in rats (Parkinson et al. 1983), AhR-dependent toxic equivalency factors were revised at an expert meeting organized by the World Health Organization (van den Berg et al. 1998). In this report, van den Berg et al. (1998) described PCB-118 as a compound with weak dioxin-like activity and PCB-126 as a congener with strong dioxin-like properties. The present findings demonstrate that an individual PCB congener known to widely contaminate human populations can alter the course of neural differentiation in primary NHNP cells. This effect was restricted to PCB-118, which has weak dioxin-like activity, and was not observed following treatment with PCB-126, a dioxin-like congener, despite the fact that these cells express the dioxin receptor (AhR). Moreover, the effect of PCB exposure on oligodendrocyte differentiation was similar to the effect of T_3_ and could be blocked by the T_3_ antagonist NH-3. Therefore, these findings suggest that nondioxin-like PCB congeners such as PCB-118 may directly interfere with TH signaling in the developing human brain, altering the course of neural differentiation and potentially accounting for the observation that exposure to PCBs is linked to cognitive deficits in the human population.

We are the first to establish a human primary cell model for investigating endocrine disruption in neural development. NHNP cells, which have the ability to differentiate into the three major cell types of the human brain—neurons, astrocytes, and oligodendrocytes (Figure 2)—formed the basis of this model. The number of oligodendrocytes was relatively low, with approximately 30% of the differentiated cells being neurons and approximately 70% appearing as astrocytes (data not shown). Other laboratories have reported a distinct distribution pattern of neurons and glia cells in human neurospheres (Buc-Caron 1995; Caldwell et al. 2001; Kanemura et al. 2002; Messina et al. 2003; Piper et al. 2001). These differences may be due to culture conditions, ages of the embryos/fetuses, or the brain areas from which the cells were prepared. Nevertheless, the low abundance of oligodendrocytes in NPHH cells provides a very sensitive system to identify agents that induce their differentiation.

Two important features of our *in vitro* model support their use in studies of chemical exposure on neurodevelopment: their xenobiotic metabolic capacity and their TH signal transduction machinery. mRNA analyses reveal that NHNP cells express a variety of phase 1 and phase 2 enzymes (Figure 3), which indicates that the cell may be capable of xenobiotic metabolism. This is important because the parent PCB congeners may be metabolized before developing toxicity (James 2001). In regard to the expression pattern of phase 1 and phase 2 enzymes, no data are available for the developing human brain. However, in adult brain, the expression of CYPs differs partially from NHNP cells (Nishimura et al. 2003); we did not identify CYP2A6 or CYP3A4 expression in NHNP cells, but adult brain exhibits a relatively high abundance of these enzymes compared with CYP1A1 expression. In contrast, neurospheres expressed CYP1A1, CYP1B1, and CYP2D6. These enzymes are also present in adult brain (Nishimura et al. 2003). Furthermore, NHNP cells express phase 2 enzymes; GSTM1 and GSTT1 were present in NHNP cells and were found in human brain tissue as well (Sherratt et al. 1997). To the contrary, human adult brain, but not NHNP cells, expressed UGT1A6 (King et al. 1999). Because of the abundance of phase 1 and phase 2 enzymes, we consider NHNP cells to be a suitable toxicologic model for studying the effects of xenobiotics on the human developing nervous system.

TH and RA are fundamental for brain development (reviewed by Bernal et al. 2003 and by McCaffery et al. 2003). They exert their actions through nuclear hormone receptors (i.e., TR, RAR, and RXR). An important premise for investigating endocrine disruption of the thyroid hormone system by PCB is expression of the involved receptors; TRα_1_, β_1_, and β_2_, as well as all RAR and RXR isoforms, with exception of RARg, were present in NHNP cells. This is in agreement with the distribution of these receptors in adult rodent brains (Zetterstrom et al. 1999). TR mRNA and protein was also detected in human fetal brain (Bernal and Pekonen 1984; Kilby et al. 2000).

In the present study, we found that the mono-*ortho*-substituted PCB-118, as well as TH, leads to an increased formation of oligodendrocytes in NHNP cells. The development of oligodendrocytes, which are the myelin producing cells in the central nervous system, is dependent on TH, which aids proliferation and survival of oligodendrocyte pre-progenitor cells (Barres et al. 1994; Ben Hur et al. 1998; Schoonover et al. 2004). The importance of TH for oligodendrocyte formation was further confirmed in hypothyroid animals exhibiting fewer numbers of oligodendrocytes than control animals (Ahlgren et al. 1997).

PCBs have been observed to have an intrinsic TH-like effect: rat pups exposed to Aroclor 1254 opened their eyes at an earlier time point, an effect that is elicited with an excess of T_4_ (Brosvic et al. 2002; Goldey et al. 1995). In addition, in pregnant animals Aroclor treatment led to an increased expression of TH-dependent genes such as RC3/neurogranin and myelin basic protein in fetal brains (Zoeller et al. 2000), although PCB can cause a decrease of serum TH levels (Gauger et al. 2004; Meerts et al. 2002; Morse et al. 1993, 1996). Most studies performed on the effects of PCBs used Aroclor, technical mixtures of PCBs containing planar and nonplanar congeners. Because of the heterogeneity of these mixtures, we decided to apply a single congener approach with two different pentachlorbiphenyls that have weak and strong dioxin-like activities, respectively. Our results show for the first time that PCB-118 exerts a TH-like effect on a cellular level in primary human cells by increasing the number of oligodendrocytes (Figure 4).

In our study of the molecular mechanism of PCB effects on oligodendrocytes, we investigated the TH-like effect of PCB-118 and whether it is mediated through the TH receptor complex. Therefore, we performed the experiments in the presence of the specific TR antagonist NH-3. NH-3 binds to the ligand-binding domain of the TRs, with selectivity for TRβ over TRα, leading to a conformational change of the receptor with release of TR corepressors. Unlike TH, NH-3 prohibits the subsequent recruitment of TR coactivators. Specificity of TRβ inhibition was shown *in vitro* and *in vivo* (Lim et al. 2002; Nguyen et al. 2002). In the presence of NH-3 the formation of oligodendrocytes by TH and PCB-118 was blocked (Figure 5A), which may indicate that the TRβ complex is involved in PCB-118–mediated effects on oligodendrocyte differentiation. Because Gauger et al. (2004) showed that a large variety of PCBs, including PCB-118, and their metabolites do not competitively bind to TR, we speculate that the TH-like effect of PCB-118 on neural differentiation is due to facilitation of coactivator binding.

In another approach to investigate whether PCB-118 acts through the TR complex, we cotreated NHNP cells with RA. As shown in Figure 5B, RA anticipated oligodendrocyte formation induced by TH or PCB-118 treatment. RA binds to the RAR receptor, which shares its heterodimerization partner RXR with several other nuclear receptors including TR (reviewed by Rowe 1997). Therefore, we suggest that antagonism of TH or PCB-118 by RA is caused by competition over RXR. A similar antagonism of TH by RA has been described by Davis and Lazar (1992), and it has been hypothesized that participation of RXR in other activation pathways may modify the cellular response to TH (Sarlieve et al. 2004).

Regarding the metabolic capacity of these progenitor cells, we cannot exclude that the observed induction of oligodendrocytes by PCB-118 is a result of PCB metabolites rather than the parent substance, and further experiments are needed. However, the observed effect is congener specific because PCB-126 did not increase oligodendrocytes in NHNP cells. PCB-126 is a coplanar biphenyl that activates the AhR, whereas PCB-118 is mono-*ortho* substituted and exerts only weak AhR agonist activity (Hestermann et al. 2000). The inability of BAP, a classical AhR agonist, to induce oligodendrocyte formation in NHNP cells (data not shown) supports the suggestion that the AhR is not involved in the disturbance of neural differentiation.

In summary, we developed a primary human *in vitro* model for investigating endocrine disruption of neural development. We identified the mono-*ortho*-substituted PCB-118 as a TH disrupter on human neural development because it induced oligodendrocyte formation in NHNP cells. In contrast, PCB-126, a coplanar AhR ligand, showed no hormone-like activity. The effects seen after PCB-118 treatment seem to be mediated through the TR complex because they can be antagonized by the TR antagonist NH-3 and by RA. The precise molecular mechanisms require further elucidation.

## Figures and Tables

**Table 1:** Sequences of oligonucleotides used to perform RT-PCRs with NHNP cells as shown in Figure 1.

Gene	Sequences	Size (bp)	Annealing temperature (°C)	Reference
β-Actin	FW CCCCAGGCACCAGGGCGTGAT RW GGTCATCTTCTCGCGGTTGGCCTTGGGGT	263	60	Ihm et al. 2002
NSE	FW CCCACTGATCCTTCCCGATACAT RW CCGATCTGGTTGACCTTGAGCA	254	60	Kukekov et al. 1999
GFAP	FW GATCAACTCACCGCCAACAGC RW CTCCTCCTCCAGCGACTCAATCT	206	60	Kukekov et al. 1999
PLP/dm20	FW CCATGCCTTCCAGTATGTCATC RW GTGGTCCAGGTGTTGAAGTAAATGT	354 PLP 249 dm20	59	Kukekov et al. 1999
CYP1A1	FW TAGACACTGATCTGGCTGCAG RW GGGAAGGCTCCATCAGCATC	146	60	Omiecinski et al. 1990
CYP1B1	FW AACGTCATGAGTGCCGTGTGT RW GGCCGGTACGTTCTCCAAATC	360	63	Sutter et al. 1994
CYP2A6	FW CAGCTGAACACAGAGCAGATGTACA RW CGCTCCCCGTTGCTGAATA	227	60	Yengi et al. 2003
CYP2B6	FW CATTCTTCCGGGGATATGGTG RW CCTCATAGTGGTCACAGAGAATCG	83	60	Yengi et al. 2003
CYP2C9	FW GAGGAGTTTTCTGGAAGAGGCAT RW CAAAATTCCGCAGCGTCAT	130	60	Yengi et al. 2003
CYP2C19	FW GAGGAGTTTTCTGGAAGAGGCC RW CATTGCTGAAAACGATTCCAAA	76	60	Yengi et al. 2003
CYP2D6	FW CTTTCTGCGCGAGGTGCT RW TGGGTCAGGAAAGCCTTTTG	96	60	Yengi et al. 2003
CYP3A4	FW TCTCATCCCAGACTTGGCCA RW CATGTGAATGGGTTCCATATAGATAGA	85	60	Yengi et al. 2003
UGT1A6	FW TCCTGGCTGAGTATTTGGGCC RW GTTCGCAAGATTCGATGGTCG	562	59	Strassburg et al. 1997
GSTM1	FW GAACTCCCTGAAAAGCTAAAGCT RW GTTGGGCTCAAATATACGGTGG	132	60	Ko et al. 2000
GSTT1	FW TTCCTTACTGGTCCTCACATCTC RW TCCCAGCTCACCGGATCAT	262	60	Ko et al. 2000
TRα1	FW CCCTGAAAACCAGCATGTCAG RW TTCTTCTGGATTGTGCGGC	150	68	Silva et al. 2002
TRβ1	FW AAGTGCCCAGACCTTCCAAA RW AAAGAAACCCTTGCAGCCTTC	150	68	Silva et al. 2002
TRβ2	FW GGGCTGGAGAATGCATGCGTAGACT RW ATTCACTGCCCAGGCCTGTTCCATA	239	68	Gittoes et al. 1997
RAR-α	FW ACCCCCTCTACCCCGCATCTACAAG RW CATGCCCACTTCAAAGCACTTCTGC	226	60	Kimura et al. 2002
RAR-β	FW ATTCCAGTGCTGACCATCGAGTCC RW CCTGTTTCTGTGTCATCCATTTCC	349	62	Kimura et al. 2002
RAR-γ	FW TACCACTATGGGGTCAGC RW CCGGTCATTTCGCACAGCT	195	60	Kimura et al. 2002
RXR-α	FW TTCGCTAAGCTCTTGCTC RW ATAAGGAAGGTGTCAATGGG	113	58	Kimura et al. 2002
RXR-β	FW GAAGCTCAGGCAAACACTAC RW TGCAGTCTTTGTTGTCCC	111	58	Kimura et al. 2002
RXR-γ	FW GCAGTTCAGAGGACATCAAGCC RW GCCTCACTCTCAGCTCGCTCTC	352	62	Kimura et al. 2002
				GenBank accession no.*^a^*/position in sequence
AhR	FW TGGTCTCCCCCAGACAGTAG RW TTCATTGCCAGAAAACCAGA	132	60	BC070080/1113-1244
AhRR	FW CAGTTACCTCCGGGTGAAGA RW CCAGAGCAAAGCCATTAAGA	161	60	NM_020731/269-429

Abbreviations: AhR, arylhydrocarbon receptor; AhRR, AhR repressor; CYP, cytochrome P450; FW, forward primer; GST, glutathione *S*-transferase; NSE, neuron specific enolase; PLP, proteolipid protein; RAR, retinoic acid receptor; RW, reverse primer; RXR, retinoic x receptor; UGT, UDP glucuronosyltransferase; TR, thyroid hormone receptor.

a GenBank (2005).

## References

[b1-ehp0113-000871] Ahlgren SC, Wallace H, Bishop J, Neophytou C, Raff MC (1997). Effects of thyroid hormone on embryonic oligodendrocyte precursor cell development in vivo and in vitro. Mol Cell Neurosci.

[b2-ehp0113-000871] Ayotte P, Muckle G, Jacobson JL, Jacobson SW, Dewailly É (2003). Assessment of pre- and postnatal exposure to polychlorinated biphenyls: lessons from the Inuit Cohort Study. Environ Health Perspect.

[b3-ehp0113-000871] Barres BA, Lazar MA, Raff MC (1994). A novel role for thyroid hormone, glucocorticoids and retinoic acid in timing oligodendrocyte development. Development.

[b4-ehp0113-000871] Barter RA, Klaassen CD (1992). UDP-glucuronosyltransferase inducers reduce thyroid hormone levels in rats by an extrathyroidal mechanism. Toxicol Appl Pharmacol.

[b5-ehp0113-000871] Ben Hur T, Rogister B, Murray K, Rougon G, Dubois-Dalcq M (1998). Growth and fate of PSA-NCAM+ precursors of the postnatal brain. J Neurosci.

[b6-ehp0113-000871] Berger DF, Lombardo JP, Jeffers PM, Hunt AE, Bush B, Casey A (2001). Hyperactivity and impulsiveness in rats fed diets supplemented with either Aroclor 1248 or PCB-contaminated St. Lawrence River fish. Behav Brain Res.

[b7-ehp0113-000871] Bernal J, Guadano-Ferraz A, Morte B (2003). Perspectives in the study of thyroid hormone action on brain development and function. Thyroid.

[b8-ehp0113-000871] Bernal J, Pekonen F (1984). Ontogenesis of the nuclear 3,5,3′-triiodothyronine receptor in the human fetal brain. Endocrinology.

[b9-ehp0113-000871] Brannen CL, Sugaya K (2000). In vitro differentiation of multi-potent human neural progenitors in serum-free medium. Neuroreport.

[b10-ehp0113-000871] Brosvic GM, Taylor JN, Dihoff RE (2002). Influences of early thyroid hormone manipulations: delays in pup motor and exploratory behavior are evident in adult operant performance. Physiol Behav.

[b11-ehp0113-000871] Brouwer A, Morse DC, Lans MC, Schuur AG, Murk AJ, Klasson-Wehler E (1998). Interactions of persistent environmental organohalogens with the thyroid hormone system: mechanisms and possible consequences for animal and human health. Toxicol Ind Health.

[b12-ehp0113-000871] Buc-Caron MH (1995). Neuroepithelial progenitor cells explanted from human fetal brain proliferate and differentiate in vitro. Neurobiol Dis.

[b13-ehp0113-000871] Caldwell MA, He X, Wilkie N, Pollack S, Marshall G, Wafford KA (2001). Growth factors regulate the survival and fate of cells derived from human neurospheres. Nat Biotechnol.

[b14-ehp0113-000871] Chana A, Concejero MA, de Frutos M, Gonzalez MJ, Herradon B (2002). Computational studies on biphenyl derivatives. Analysis of the conformational mobility, molecular electrostatic potential, and dipole moment of chlorinated biphenyl: searching for the rationalization of the selective toxicity of polychlorinated biphenyls (PCBs). Chem Res Toxicol.

[b15-ehp0113-000871] Darvill T, Lonky E, Reihman J, Stewart P, Pagano J (2000). Prenatal exposure to PCBs and infant performance on the Fagan Test of Infant Intelligence. Neurotoxicology.

[b16-ehp0113-000871] Davis KD, Lazar MA (1992). Selective antagonism of thyroid hormone action by retinoic acid. J Biol Chem.

[b17-ehp0113-000871] DeKoning EP, Karmaus W (2000). PCB exposure in utero and via breast milk. A review. J Expo Anal Environ Epidemiol.

[b18-ehp0113-000871] Döhr O, Vogel C, Abel J (1995). Different response of 2,3,7,8-tetrachlorodibenzo-*p*-dioxin (TCDD)-sensitive genes in human breast cancer MCF-7 and MDA-MB 231 cells. Arch Biochem Biophys.

[b19-ehp0113-000871] Dowling AL, Zoeller RT (2000). Thyroid hormone of maternal origin regulates the expression of RC3/neurogranin mRNA in the fetal rat brain. Brain Res Mol Brain Res.

[b20-ehp0113-000871] Gauger KJ, Kato Y, Haraguchi K, Lehmler HJ, Robertson LW, Bansal R (2004). Polychlorinated biphenyls (PCBs) exert thyroid hormone-like effects in the fetal rat brain but do not bind to thyroid hormone receptors. Environ Health Perspect.

[b21-ehp0113-000871] GenBank2005. GenBank Overview. Bethesda, MD:National Center for Biotechnology Information, National Library of Medicine. Available: http://www.ncbi.nlm.nih.gov/Genbank/GenbankOverview.html [accessed 26 May 2005].

[b22-ehp0113-000871] Gittoes NJ, McCabe CJ, Verhaeg J, Sheppard MC, Franklyn JA (1997). Thyroid hormone and estrogen receptor expression in normal pituitary and nonfunctioning tumors of the anterior pituitary. J Clin Endocrinol Metab.

[b23-ehp0113-000871] Goldey ES, Kehn LS, Lau C, Rehnberg GL, Crofton KM (1995). Developmental exposure to polychlorinated biphenyls (Aroclor 1254) reduces circulating thyroid hormone concentrations and causes hearing deficits in rats. Toxicol Appl Pharmacol.

[b24-ehp0113-000871] Haddow JE, Palomaki GE, Allan WC, Williams JR, Knight GJ, Gagnon J (1999). Maternal thyroid deficiency during pregnancy and subsequent neuropsychological development of the child. N Engl J Med.

[b25-ehp0113-000871] Hagmar L (2003). Polychlorinated biphenyls and thyroid status in humans: a review. Thyroid.

[b26-ehp0113-000871] Haraguchi K, Kato Y, Kimura R, Masuda Y (1997). Comparative study on formation of hydroxy and sulfur-containing metabolites from different chlorinated biphenyls with 2,5-substitution in rats. Drug Metab Dispos.

[b27-ehp0113-000871] Harry GJ, Billingsley M, Bruinink A, Campbell IL, Classen W, Dorman DC (1998). *In vitro* techniques for the assessment of neurotoxicity. Environ Health Perspect.

[b28-ehp0113-000871] Hestermann EV, Stegeman JJ, Hahn ME (2000). Relative contributions of affinity and intrinsic efficacy to aryl hydrocarbon receptor ligand potency. Toxicol Appl Pharmacol.

[b29-ehp0113-000871] Huisman M, Koopman-Esseboom C, Fidler V, Hadders-Algra M, van der Paauw CG, Tuinstra LG (1995a). Perinatal exposure to polychlorinated biphenyls and dioxins and its effect on neonatal neurological development. Early Hum Dev.

[b30-ehp0113-000871] Huisman M, Koopman-Esseboom C, Lanting CI, van der Paauw CG, Tuinstra LG, Fidler V (1995b). Neurological condition in 18-month-old children perinatally exposed to polychlorinated biphenyls and dioxins. Early Hum Dev.

[b31-ehp0113-000871] Ihm CG, Park JK, Kim HJ, Lee TW, Cha DR (2002). Effects of high glucose on interleukin-6 production in human mesangial cells. J Korean Med Sci.

[b32-ehp0113-000871] James JO2001. Polychlorinated biphenyls: metabolism and metabolites. In: PCBs-Recent Advances in Environmental Toxicology and Health Effects (Robertson LW, Hansen LG, eds). Lexington, KY:University Press of Kentucky, 35–46.

[b33-ehp0113-000871] Kanemura Y, Mori H, Kobayashi S, Islam O, Kodama E, Yamamoto A (2002). Evaluation of in vitro proliferative activity of human fetal neural stem/progenitor cells using indirect measurements of viable cells based on cellular metabolic activity. J Neurosci Res.

[b34-ehp0113-000871] Kilby MD, Gittoes N, McCabe C, Verhaeg J, Franklyn JA (2000). Expression of thyroid receptor isoforms in the human fetal central nervous system and the effects of intrauterine growth restriction. Clin Endocrinol (Oxf).

[b35-ehp0113-000871] Kim M, Kim S, Yun S, Lee M, Cho B, Park J (2004). Comparison of seven indicator PCBs and three coplanar PCBs in beef, pork, and chicken fat. Chemosphere.

[b36-ehp0113-000871] Kimura Y, Suzuki T, Kaneko C, Darnel AD, Moriya T, Suzuki S (2002). Retinoid receptors in the developing human lung. Clin Sci (Lond).

[b37-ehp0113-000871] King CD, Rios GR, Assouline JA, Tephly TR (1999). Expression of UDP-glucuronosyltransferases (UGTs) 2B7 and 1A6 in the human brain and identification of 5-hydroxytryptamine as a substrate. Arch Biochem Biophys.

[b38-ehp0113-000871] Ko Y, Koch B, Harth V, Sachinidis A, Thier R, Vetter H (2000). Rapid analysis of GSTM1, GSTT1 and GSTP1 polymorphisms using real-time polymerase chain reaction. Pharmacogenetics.

[b39-ehp0113-000871] Kolaja KL, Klaassen CD (1998). Dose-response examination of UDP-glucuronosyltransferase inducers and their ability to increase both TGF-beta expression and thyroid follicular cell apoptosis. Toxicol Sci.

[b40-ehp0113-000871] Konig S, Moura Neto V (2002). Thyroid hormone actions on neural cells. Cell Mol Neurobiol.

[b41-ehp0113-000871] Kukekov VG, Laywell ED, Suslov O, Davies K, Scheffler B, Thomas LB (1999). Multipotent stem/progenitor cells with similar properties arise from two neurogenic regions of adult human brain. Exp Neurol.

[b42-ehp0113-000871] Lilienthal H, Neuf M, Munoz C, Winneke G (1990). Behavioral effects of pre- and postnatal exposure to a mixture of low chlorinated PCBs in rats. Fundam Appl Toxicol.

[b43-ehp0113-000871] Lim W, Nguyen NH, Yang HY, Scanlan TS, Furlow JD (2002). A thyroid hormone antagonist that inhibits thyroid hormone action in vivo. J Biol Chem.

[b44-ehp0113-000871] McCaffery PJ, Adams J, Maden M, Rosa-Molinar E (2003). Too much of a good thing: retinoic acid as an endogenous regulator of neural differentiation and exogenous teratogen. Eur J Neurosci.

[b45-ehp0113-000871] McKinney JD, Waller CL (1998). Molecular determinants of hormone mimicry: halogenated aromatic hydrocarbon environmental agents. J Toxicol Environ Health B Crit Rev.

[b46-ehp0113-000871] Meerts IA, Assink Y, Cenijn PH, Van Den Berg JH, Weijers BM, Bergman A (2002). Placental transfer of a hydroxylated polychlorinated biphenyl and effects on fetal and maternal thyroid hormone homeostasis in the rat. Toxicol Sci.

[b47-ehp0113-000871] Messina DJ, Alder L, Tresco PA (2003). Comparison of pure and mixed populations of human fetal-derived neural progenitors transplanted into intact adult rat brain. Exp Neurol.

[b48-ehp0113-000871] Morreale de Escobar G, Obregón MJ, Escobar del Rey F (2000). Is neuropsychological development related to maternal hypothyroidism or to maternal hypothyroxinemia?. J Clin Endocrinol Metab.

[b49-ehp0113-000871] Morse DC, Groen D, Veerman M, van Amerongen CJ, Koeter HB, Smits van Prooije AE (1993). Interference of polychlorinated biphenyls in hepatic and brain thyroid hormone metabolism in fetal and neonatal rats. Toxicol Appl Pharmacol.

[b50-ehp0113-000871] Morse DC, Wehler EK, Wesseling W, Koeman JH, Brouwer A (1996). Alterations in rat brain thyroid hormone status following pre- and postnatal exposure to polychlorinated biphenyls (Aroclor 1254). Toxicol Appl Pharmacol.

[b51-ehp0113-000871] Nagayama J, Tsuji H, Iida T, Hirakawa H, Matsueda T, Ohki M (2001). Effects of contamination level of dioxins and related chemicals on thyroid hormone and immune response systems in patients with “Yusho. Chemosphere.

[b52-ehp0113-000871] Nguyen NH, Apriletti JW, Cunha Lima ST, Webb P, Baxter JD, Scanlan TS (2002). Rational design and synthesis of a novel thyroid hormone antagonist that blocks coactivator recruitment. J Med Chem.

[b53-ehp0113-000871] Nishimura M, Yaguti H, Yoshitsugu H, Naito S, Satoh T (2003). Tissue distribution of mRNA expression of human cytochrome P450 isoforms assessed by high-sensitivity real-time reverse transcription PCR. Yakugaku Zasshi.

[b54-ehp0113-000871] Omiecinski CJ, Redlich CA, Costa P (1990). Induction and developmental expression of cytochrome P450IA1 messenger RNA in rat and human tissues: detection by the polymerase chain reaction. Cancer Res.

[b55-ehp0113-000871] Osius N, Karmaus W, Kruse H, Witten J (1999). Exposure to polychlorinated biphenyls and levels of thyroid hormones in children. Environ Health Perspect.

[b56-ehp0113-000871] Parkinson A, Safe SH, Robertson LW, Thomas PE, Ryan DE, Reik LM (1983). Immunochemical quantitation of cytochrome P-450 isozymes and epoxide hydrolase in liver microsomes from polychlorinated or polybrominated biphenyl-treated rats. A study of structure-activity relationships. J Biol Chem.

[b57-ehp0113-000871] Piper DR, Mujtaba T, Keyoung H, Roy NS, Goldman SA, Rao MS (2001). Identification and characterization of neuronal precursors and their progeny from human fetal tissue. J Neurosci Res.

[b58-ehp0113-000871] Pop VJ, Kuijpens JL, van Baar AL, Verkerk G, van Son MM, de Vijlder JJ (1999). Low maternal free thyroxine concentrations during early pregnancy are associated with impaired psychomotor development in infancy. Clin Endocrinol (Oxf).

[b59-ehp0113-000871] Porterfield SP (2000). Thyroidal dysfunction and environmental chemicals—potential impact on brain development. Environ Health Perspect.

[b60-ehp0113-000871] Porterfield SP, Hendry LB (1998). Impact of PCBs on thyroid hormone directed brain development. Toxicol Ind Health.

[b61-ehp0113-000871] Roegge CS, Seo BW, Crofton KM, Schantz SL (2000). Gestational-lactational exposure to Aroclor 1254 impairs radial-arm maze performance in male rats. Toxicol Sci.

[b62-ehp0113-000871] Rowe A (1997). Retinoid X receptors. Int J Biochem Cell Biol.

[b63-ehp0113-000871] Sarlieve LL, Rodriguez-Pena A, Langley K (2004). Expression of thyroid hormone receptor isoforms in the oligodendrocyte lineage. Neurochem Res.

[b64-ehp0113-000871] Schantz SL, Widholm JJ, Rice DC (2003). Effects of PCB exposure on neuropsychological function in children. Environ Health Perspect.

[b65-ehp0113-000871] Schell L DeCaprio A Gallo M Hubicki L The Akwesasne Task Force on the Environment2002. Polychlorinated biphenyls and thyroid function in adolescents of the Mohawk Nation at Akwesasne. In: Human Growth from Conception to Maturity (Gilli G, Schell L, Benso L, eds). London:Smith-Gordon, 289–296.

[b66-ehp0113-000871] Schoonover CM, Seibel MM, Jolson DM, Stack MJ, Rahman RJ, Jones SA (2004). Thyroid hormone regulates oligodendrocyte accumulation in developing rat brain white matter tracts. Endocrinology.

[b67-ehp0113-000871] Sherratt PJ, Pulford DJ, Harrison DJ, Green T, Hayes JD (1997). Evidence that human class Theta glutathione *S*-transferase T1-1 can catalyse the activation of dichloromethane, a liver and lung carcinogen in the mouse. Comparison of the tissue distribution of GST T1-1 with that of classes Alpha, Mu and Pi GST in human. Biochem J.

[b68-ehp0113-000871] Silva JM, Dominguez G, Gonzalez-Sancho JM, Garcia JM, Silva J, Garcia-Andrade C (2002). Expression of thyroid hormone receptor/erbA genes is altered in human breast cancer. Oncogene.

[b69-ehp0113-000871] Strassburg CP, Oldhafer K, Manns MP, Tukey RH (1997). Differential expression of the UGT1A locus in human liver, biliary, and gastric tissue: identification of UGT1A7 and UGT1A10 transcripts in extrahepatic tissue. Mol Pharmacol.

[b70-ehp0113-000871] Sutter TR, Tang YM, Hayes CL, Wo YYP, Jabs EW, Li X (1994). Complete cDNA sequence of a human dioxin-inducible mRNA identifies a new gene subfamily of cytochrome P450 that maps to chromosome 2. J Biol Chem.

[b71-ehp0113-000871] Svendsen CN, ter Borg MG, Armstrong RJ, Rosser AE, Chandran S, Ostenfeld T (1998). A new method for the rapid and long term growth of human neural precursor cells. J Neurosci Methods.

[b72-ehp0113-000871] Tilson HA (1996). Evolution and current status of neurotoxicity risk assessment. Drug Metab Rev.

[b73-ehp0113-000871] van den Berg M, Birnbaum L, Bosveld AT, Brunstrom B, Cook P, Feeley M (1998). Toxic equivalency factors (TEFs) for PCBs, PCDDs, PCDFs for humans and wildlife. Environ Health Perspect.

[b74-ehp0113-000871] Walkowiak J, Wiener JA, Fastabend A, Heinzow B, Kramer U, Schmidt E (2001). Environmental exposure to polychlorinated biphenyls and quality of the home environment: effects on psychodevelopment in early childhood. Lancet.

[b75-ehp0113-000871] Widholm JJ, Clarkson GB, Strupp BJ, Crofton KM, Seegal RF, Schantz SL (2001). Spatial reversal learning in Aroclor 1254-exposed rats: sex-specific deficits in associative ability and inhibitory control. Toxicol Appl Pharmacol.

[b76-ehp0113-000871] Yengi LG, Xiang Q, Pan J, Scatina J, Kao J, Ball SE (2003). Quantitation of cytochrome P450 mRNA levels in human skin. Anal Biochem.

[b77-ehp0113-000871] Zetterstrom RH, Lindqvist E, Mata de Urquiza A, Tomac A, Eriksson U, Perlmann T (1999). Role of retinoids in the CNS: differential expression of retinoid binding proteins and receptors and evidence for presence of retinoic acid. Eur J Neurosci.

[b78-ehp0113-000871] Zoeller RT, Crofton KM (2000). Thyroid hormone action in fetal brain development and potential for disruption by environmental chemicals. Neurotoxicology.

[b79-ehp0113-000871] Zoeller RT, Dowling AL, Vas AA (2000). Developmental exposure to polychlorinated biphenyls exerts thyroid hormone-like effects on the expression of RC3/neurogranin and myelin basic protein messenger ribonucleic acids in the developing rat brain. Endocrinology.

